# Intakes of energy, macronutrients, and micronutrients in adult Lithuanian population: a national study of 2019–2020

**DOI:** 10.1017/jns.2024.40

**Published:** 2024-09-23

**Authors:** Gabija Bulotaitė, Roma Bartkevičiūtė, Albertas Barzda, Rimantas Stukas

**Affiliations:** 1 Department of Public Health, Institute of Health Sciences, Faculty of Medicine, Vilnius University, Vilnius, Lithuania; 2 Institute of Hygiene, Vilnius, Lithuania

**Keywords:** Dietary survey, Energy intake, Food consumption survey, Nutrient intake

## Abstract

Dietary surveys are critical in evaluating dietary trends prevailing across the entire population. The aim of this study is to present the results of the latest research into the intakes of energy, macronutrients, and micronutrients amongst the adult population in Lithuania from 2019 through 2020. A cross-sectional study was conducted and dietary data was collected using a 24-h dietary recall method from a total of 2555 Lithuanian adults. Medians, 25^th^ and 75^th^ percentiles were calculated. The diet of Lithuanian adults was found insufficient as the energy intake from fats exceeded the recommended norms, while the energy intake from carbohydrates was below the lower range. The intakes of dietary fibre and most vitamins and minerals were insufficient. Men, compared to women, had a statistically higher total energy intake and energy intake from fats, and a higher intake of dietary fibre. Younger adults, compared to older ones, had statistically lower intakes of energy from fats, including saturated fats, and lower intakes of sodium chloride. Respondents with primary education, compared to those who had attained a higher degree of education, had a statistically lower intake of total energy and dietary fibre. Study showed that intakes of majority of nutrients in the diet of the adult Lithuanian population are not in compliance with the recommended daily intakes. Continuous nationally representative studies into food consumption and nutrient intake of the adult Lithuanian population must be carried out to assess the changes in the population’s diet and the effectiveness of policies aimed at promoting healthy diets.

## Introduction

Although the life expectancy of the population in Lithuania has increased in recent years, it still remains among the shortest in the European Union (EU) and the national preventable mortality rate is significantly higher than the EU average.^(1)^ Unhealthy nutrition is recognized as one of the preventable risk factors for health. Furthermore, scientific evidence shows that changing one’s diet can have significant, both positive and negative, lifelong effects on human health. The consumption of foods high in fat, especially saturated fats and trans fatty acids, sugars, sodium can have a negative effect on health, while the consumption of products high in dietary fibres such as vegetables, fruits, legumes, whole grain products, fish, vitamins is beneficial for health.^(2)^ However, the studies of food and nutrient intake carried out in different European countries, including Lithuania, show that the diet of European residents is still insufficient in nutrients. The intakes of sugars, fats, saturated fats, sodium exceed the recommended norms, however, the intake of dietary fibre is insufficient.^(3)^

Dietary surveys are critical in evaluating dietary trends prevailing across the entire population. The importance of nationally representative diet and nutrition surveys is highlighted in the WHO’s European Food and Nutrition Action Plan, as well as in the Lithuanian Health Strategy for 2014–2025 adopted in 2014.^(4,5)^ These and similar surveys provide essential information about dietary trends of the population, facilitate the identification of inequalities and are important tools in evaluating policies aimed at promoting healthy diets.^(2,6)^

The first nationally representative study of food consumption and nutrient intake of the adult Lithuanian population was carried out in 1997, subsequent studies were carried out in 2007, 2013 and 2019–2020. The aim of this article is to present the results of the intakes of energy, macronutrients and micronutrients in the adult Lithuanian population obtained from the latest study carried out in 2019–2020.

## Methods

### Study population

Based on the latest Lithuanian census data provided by the State Data Agency of Lithuania, a sample of the local adult population was defined to represent the adults of Lithuania based on gender, age, and other socio-demographic characteristics. The current study on food consumption and dietary intake of Lithuanian adults was completed over the period of 2019-2020 and is part of the national study initiated by the Ministry of Health of the Republic of Lithuania in cooperation with Vilnius University and being sustainably carried out into the actual nutrition, dietary and physical activity habits and knowledge of nutrition and physical activity of the Lithuanian adults and elderly.

### Dietary intake

The assessment of dietary intake of energy and nutrients was conducted using a single 24-h dietary recall. The data was collected using a face-to-face interview. Interviewers were trained prior to the interviews. Average duration of an interview was 30 minutes. An atlas with photos of commonly consumed foods and their portion sizes was used to record the data on food products and their quantities consumed by each respondent during the previous day. The intakes of energy, macronutrients and micronutrients were estimated using nutritional software which contains a food composition database supplemented with the composition of food products and dishes which are typically consumed in Lithuania.

Nutrient intakes of the adults were compared with the recommended daily intakes (RDI), which have been approved by the order of the Minister of Health of the Republic of Lithuania.^(7)^ These recommended daily intakes are listed in Table 1.

**Table 1.** Recommended daily intakes (RDI) of nutrients for Lithuanian adults

Nutrient	RDI	Vitamins	RDI	Minerals	RDI
Proteins (E%)	10–20	Vitamin A (μg RE/day)	800	Magnesium (mg/day)	325
Fats (E%)	25–35	Vitamin D (μg/day)	10	Phosphorus (mg/day)	700
Saturated fats (E%)	<10	Vitamin E (mg α-TE/day)	11	Potassium (mg/day)	3300
Carbohydrates (E%)	45–60	Vitamin K (μg/day)	75	Calcium (mg/day)	900
Sugars^a^ (E%)	<10	Thiamine (vitamin B1) (mg/day)	1.2	Chromium (μg/day)	40
Dietary fibre^b^ (g/day)	25–35	Riboflavin (vitamin B2) (mg/day)	1.4	Manganese (mg/day)	3.0
		Vitamin B6 (mg/day)	1.4	Iron (mg/day)	12.5
		Vitamin B12 (μg/day)	3.0	Copper (mg/day)	1.0
		Niacin (PP) (mg NE/day)	17	Zinc (mg/day)	10
		Vitamin C (mg/day)	80	Selenium (μg/day)	55
		Pantothenic acid (vitamin B5) (mg/day)	6.0	Iodine (μg/day)	150
		Biotin (vitamin B7) (μg/day)	50	Sodium chloride (g/day)	5–6

a Total sugar intake.

b Total daily dietary fibre intake.

### Statistical analysis

The Kolmogorov-Smirnov test was used to examine if variables were normally distributed. Since all the variables were not normally distributed, the Mann-Whitney and Kruskal-Wallis non-parametric tests were used for conducting a comparison between groups. Medians, the 25^th^ and 75^th^ percentiles were calculated for the individual intake of energy, macronutrients and micronutrients categorized by age, gender, living area, and degree of education. All statistical analyses were performed using the SPSS software package for Windows (version 20).

### Ethics statement

This study was conducted according to the guidelines laid down in the Declaration of Helsinki, all individual participants provided verbal informed consent. Verbal consent was witnessed and formally recorded. No approval from the Lithuanian Bioethics Committee was required because only anonymized data were used and the research did not collect health data, according to the Recommendations of the Board of the Lithuanian Bioethics Committee ‘Compliance with ethical principles in non-biomedical research involving human health’.

## Results

### Descriptive statistics

A total of 2555 respondents were surveyed. Women comprised 53.8% of the sample. All the subjects were grouped into the aged 19–34 representing 36.5% of the respondents, aged 35–49 involving 34.1% of the respondents, and aged 50–64 representing 29.4% of the respondents. In all, 78.5% of respondents lived in urban areas while 21.5% in rural areas.

### Total energy and macronutrient intake

The median daily energy intake for the entire adult Lithuanian population was determined to be 1691 kcal. In terms of the contribution of macronutrients to dietary energy, fats contributed the highest proportion (42.7%) followed by carbohydrates (41.1%) and proteins (14.7%). The actual median intake of protein (14.7 E%) in the diet of Lithuanian adult population was determined to be in compliance with the recommended intake which is 10–20% of the total energy intake (E%). The recommended energy intake from fats for Lithuanian adults should be between 25 and 35 E%. However, our study showed that the fat intake of the adult Lithuanian population (42.7 E%) exceeded the recommended amount. Study also showed that the intake of saturated fats in the diet of Lithuanian adults was above the recommended amount of 10 E%, since the actual median energy intake from saturated fats was determined to be 13.7 E%. It is recommended that for adults carbohydrates should provide 45–60 E%. However, according to our study, the intake of carbohydrates in the adult Lithuanian population was below the lower range as carbohydrates provided 41.1 E%. Our study has demonstrated that the median energy intake from sugars in the diet of Lithuanian adults is 9.8 E% and this is in line with the recommended intake because energy intake from sugars for Lithuanian adults be below 10 E%. The estimated daily intake of total dietary fibre was 15.1 g which is significantly lower than the recommended intake of 25–35 g of dietary fibre per day.

Table 2 shows the total energy intake, the contribution of macronutrients to the total energy intake and the dietary fibre intake according to respondents’ gender and age. Men had statistically higher total energy intake than women. The energy intake from fats, including saturated fats, was statistically higher for men than women. The energy intake from carbohydrates, including sugars, was statistically higher for women compared to men. The comparison of the energy contribution of different macronutrients according to respondents’ age showed that fat intake, including saturated fats, was statistically lower for the youngest group (aged 19–34) of adults than for older adults. Men, compared to women, and older adults compared to the youngest group of adults, had a statistically higher intake of dietary fibre.

**Table 2.** Daily energy and macronutrient intakes by gender and age group (median (25^th^ and 75^th^ percentiles))

Macronutrients	Total	Gender			Age group (years)			
		Men	Women	*P-value*	19–34	35–49	50–64	*P-value*
Energy (kcal/day)	1691 (1263–2237)	1956 (1471–2585)	1492 (1148–1948)	<0.0001	1672 (1249–2241)	1700 (1269–2317)	1707 (1267–2199)	0.552
Proteins (E%)	14.7 (12.4–18.2)	14.6 (12.4–17.8)	14.7 (12.4–18.5)	0.391	14.6 (12.1–18.1)	14.7 (12.4–18.3)	14.7 (12.5–18.0)	0.615
Fats (E%)	42.7 (35.3–50.4)	43.5 (36.3–50.9)	41.9 (34.6–50.1)	<0.0001	41.6 (34.4–49.3)	43.2 (36.4–50.7)	43.2 (35.3–51.0)	0.002
Saturated fats (E%)	13.7 (11.3–16.1)	13.9 (11.6–16.3)	13.4 (11.1–16.0)	<0.0001	13.3 (11.0–15.8)	13.8 (11.7–16.2)	13.8 (11.3–16.3)	0.002
Carbohydrates (E%)	41.1 (33.1–49.4)	40.2 (32.6–47.9)	41.7 (33.6–50.5)	<0.0001	41.8 (33.9–50.8)	40.6 (32.9–48.1)	40.6 (32.7–49.5)	0.001
Sugars^a^ (E%)	9.8 (6.0–14.2)	8.8 (5.3–12.9)	10.8 (6.9–15.3)	<0.0001	9.7 (6.2–14.4)	9.9 (6.0–14.0)	9.6 (5.8–14.1)	0.938
Dietary fibre^b^ (g/day)	15.1 (10.2–21.3)	16.2 (11.1–22.9)	14.4 (10.0–19.6)	<0.0001	14.4 (9.4–20.5)	15.3 (10.5–21.8)	15.6 (11.0–22.1)	0.001

a Total sugar intake.

b Total dietary fibre intake.

Table 3 shows the results of the total energy and macronutrient intakes according to the respondents’ living area and degree of education. While analysing the differences in macronutrient contribution to the total energy intake with respect to the respondents’ living area, it was found that the respondents who lived in urban areas had a statistically higher energy intake from sugars. The respondents with primary education, compared to respondents who had attained a higher degree of education, had statistically lower intakes of total energy and dietary fibre.

**Table 3.** Daily energy and macronutrient intakes by the living area and degree of education (median (25^th^ and–75 percentiles))

Macronutrients	Living area			Degree of education				
	Urban	Rural	*P-value*	Primary	Secondary	College, vocational	Tertiary	*P-value*
Energy (kcal/day)	1689 (1251–2312)	1693 (1254–2317)	0.744	1590 (1245–1908)	1693 (1253–2303)	1767 (1329–2347)	1663 (1258–2181)	0.004
Proteins (E%)	14.6 (12.3–18.2)	14.7 (12.6–18.1)	0.777	14.3 (12.6–17.5)	14.4 (12.1–18.3)	14.7 (12.5–17.9)	14.7 (12.4–18.2)	0.503
Fats (E%)	42.6 (35.3–50.4)	43.3 (35.5–50.8)	0.201	39.8 (34.6–49.8)	43.3 (35.8–50.8)	42.9 (35.6–50.3)	42.3 (35.1–50.4)	0.325
Saturated fats (E%)	13.6 (11.3–16.1)	13.8 (11.4–16.2)	0.231	12.7 (11.1–15.9)	13.8 (11.4–16.2)	13.7 (11.4–16.1)	13.5 (11.2–16.1)	0.325
Carbohydrates (E%)	41.1 (33.4– 49.4)	40.8 (32.3–49.1)	0.225	45.9 (34.0–50.8)	40.6 (33.2–49.4)	40.8 (33.8–48.6)	41.4 (32.5–49.7)	0.683
Sugars^a^ (E%)	10.0 (6.2–14.3)	8.9 (5.5–13.8)	0.004	9.2 (6.2–13.6)	8.9 (5.6–13.6)	9.7 (5.9–14.0)	10.3 (6.4–14.7)	0.062
Dietary fibre^b^ (g/day)	15.1 (10.3–21.1)	14.8 (9.9–22.1)	0.701	13.0 (9.7–20.7)	14.3 (9.2–20.8)	16.1 (11.0–20.7)	15.0 (10.3–20.7)	0.001

a Total sugar intake.

b Total dietary fibre intake.

### Vitamin intake

The study showed that the intakes of vitamin A, vitamin E, thiamine, riboflavin, vitamin B6, and vitamin B12 in the diet of Lithuanian adult population were in adherence or almost in adherence to the recommended daily intakes. However, study found inadequate intakes of vitamin K, vitamin D, niacin, vitamin C, pantothenic acid, and biotin.

Table 4 shows the results of the total vitamin intakes and the intakes according to respondents’ gender and age. For all vitamins, with the exception of vitamin K, men tended to have a statistically higher intake than women. Significant differences were found between the age groups for the intake of vitamin K, thiamine, and vitamin C – the respondents from the youngest age group, compared to older respondents, had a statistically lower intake of these vitamins.

**Table 4.** Daily vitamin intakes by gender and age group (median (25^th^ and 75^th^ percentiles))

Vitamins	Total	Gender			Age group (years)			
		Men	Women	*P-value*	19–34	35–49	50–64	*P-value*
Vitamin A (μg RE/day)	718.5 (438.0–1278.5)	787.7 (477.4–1400.4)	675.4 (408.1–1152.3)	<0.0001	690.6 (419.2–1258.4)	731.8 (449.2–1289.5)	757.6 (464.8–1297.5)	0.098
Vitamin D (μg/day)	5.2 (3.9–8.3)	5.6 (4.1–9.1)	4.9 (3.8–7.7)	<0.0001	5.2 (4.0–8.3)	5.2 (3.9–8.2)	5.2 (3.9–8.4)	0.853
Vitamin E (mg α-TE/day)	11.1 (6.7–18.6)	12.2 (7.6–19.8)	10.3 (6.3–17.6)	<0.0001	10.5 (6.4–18.0)	11.4 (6.9–18.8)	11.4 (6.9–19.2)	0.106
Vitamin K (μg/day)	49.1 (18.9–108.2)	50.9 (19.3–108.0)	45.8 (18.5–108.3)	0.183	40.7 (15.5–97.0)	51.6 (20.4–117.2)	51.5 (21.6–112.8)	<0.0001
Thiamine (vitamin B1) (mg/day)	1.0 (0.7–1.5)	1.2 (0.8–1.7)	0.9 (0.6–1.3)	<0.0001	1.0 (0.6–1.4)	1.1 (0.7–1.6)	1.1 (0.7–1.6)	<0.0001
Riboflavin (vitamin B2) (mg/day)	1.2 (0.8–1.6)	1.3 (0.9–1.7)	1.1 (0.8–1.4)	<0.0001	1.1 (0.8–1.5)	1.2 (0.9–1.6)	1.2 (0.9–1.6)	0.103
Vitamin B6 (mg/day)	1.5 (1.1–2.2)	1.7 (1.2–2.4)	1.4 (1.0–2.0)	<0.0001	1.6 (1.1–2.2)	1.6 (1.0–2.2)	1.5 (1.1–2.2)	0.987
Vitamin B12 (μg/day)	2.7 (1.1–4.8)	3.0 (1.1–5.3)	2.5 (1.1–4.4)	<0.0001	2.7 (1.2–4.7)	2.8 (1.1–4.9)	2.6 (1.1–4.8)	0.530
Niacin (PP) (mg NE/day)	12.1 (8.0–18.1)	14.2 (9.7–20.6)	10.5 (7.0–15.9)	<0.0001	11.7 (7.8–18.2)	12.4 (8.1–18.4)	12.3 (8.3–17.8)	0.659
Vitamin C (mg/day)	57.5 (33.8–90.4)	60.2 (35.9–93.5)	54.4 (32.6–87.2)	0.010	53.3 (31.7–88.0)	59.8 (35.1–93.1)	58.6 (34.5–90.8)	0.018
Pantothenic acid (vitamin B5) (mg/day)	2.9 (2.0–4.1)	3.3 (2.3–4.7)	2.6 (1.8–3.7)	<0.0001	2.9 (1.9–4.0)	2.9 (2.0–4.2)	2.9 (2.0–4.2)	0.543
Biotin (vitamin B7) (μg/day)	17.8 (11.3–27.9)	20.9 (13.6–31.7)	15.7 (10.1–24.7)	<0.0001	17.5 (11.0–28.0)	18.1 (11.2–27.9)	18.1 (11.5–27.9)	0.715

Table 5 shows the results of vitamin intake according to the respondents’ living area and degree of education. No significant differences were observed in vitamin intake between urban and rural respondents. The intake of pantothenic acid statistically differed in different groups by degree of education. The respondents who had attained a primary degree of education had a lower intake of pantothenic acid, compared to those, who had attained a higher degree of education.

**Table 5.** Daily vitamins intakes by the living area and degree of education (median (25^th^ and 75^th^ percentiles))

Vitamins	Living area			Degree of education				
	Urban	Rural	*P-value*	Primary	Secondary	College, vocational	Tertiary	*P-value*
Vitamin A (μg RE/day)	713.3 (438.1–1264.0)	740.0 (432.5–1301.4)	0.646	682.0 (406.9–1703.0)	671.9 (398.8–1264.0)	780.6 (482.0–1301.4)	711.8 (430.6–1257.4)	0.054
Vitamin D (μg/day)	5.1 (3.8–8.1)	5.7 (4.4–9.2)	0.311	2.6 (2.0–4.2)	4.9 (3.7–7.7)	5.2 (4.0–8.5)	5.4 (4.0–8.5)	0.271
Vitamin E (mg α-TE/day)	11.1 (6.7–18.4)	11.3 (6.5–19.3)	0.562	8.6 (6.1–17.1)	10.2 (6.5–18.2)	11.8 (7.0–19.2)	11.1 (6.5–18.5)	0.140
Vitamin K (μg/day)	48.9 (19.2–106.2)	49.5 (18.2–112.6)	0.779	39.8 (12.3–91.6)	44.7 (16.3–94.1)	52.6 (21.4–118.9)	47.7 (19.6–105.1)	0.075
Thiamine (vitamin B1) (mg/day)	1.0 (0.7–1.5)	1.1 (0.7–1.6)	0.191	1.0 (0.6–1.4)	1.1 (0.7–1.5)	1.1 (0.7–1.6)	1.0 (0.7–1.4)	0.0005
Riboflavin (vitamin B2) (mg/day)	1.2 (0.8–1.6)	1.2 (0.8–1.7)	0.527	1.0 (0.7–1.4)	1.1 (0.8–1.6)	1.2 (0.9–1.6)	1.1 (0.8–1.5)	0.091
Vitamin B6 (mg/day)	1.5 (1.1–2.2)	1.5 (1.0–2.3)	0.653	1.4 (0.8–2.1)	1.5 (1.0–2.2)	1.6 (1.1–2.3)	1.5 (1.1–2.2)	0.199
Vitamin B12 (μg/day)	2.7 (1.1–4.8)	2.7 (1.1–4.8)	0.496	2.3 (1.5–4.4)	2.5 (0.9–4.7)	2.7 (1.1–4.9)	2.8 (1.2–4.8)	0.863
Niacin (PP) (mg NE/day)	12.1 (8.1–17.9)	12.2 (7.7–19.3)	0.766	10.5 (6.5–16.2)	12.7 (8.2–18.9)	12.4 (8.5–17.8)	11.7 (7.8–18.1)	0.381
Vitamin C (mg/day)	58.0 (34.3–91.3)	54.1 (32.4–87.2)	0.092	42.4 (27.7–69.4)	52.7 (31.5–88.9)	59.8 (36.6–92.9)	58.5 (34.5–89.5)	0.062
Pantothenic acid (vitamin B5) (mg/day)	2.9 (2.0–4.1)	2.8 (1.0–4.3)	0.554	2.0 (1.5–3.3)	2.9 (1.9–4.2)	3.1 (2.2–4.3)	2.9 (1.0–4.0)	0.003
Biotin (vitamin B7) (μg/day)	17.8 (11.5–27.3)	17.9 (10.4–29.9)	0.917	12.4 (9.9–25.0)	18.1 (10.8–29.2)	18.6 (11.5–28.0)	17.5 (11.3–27.4)	0.269

### Mineral intake

According to our study, the intakes of magnesium, manganese, and zinc in the diet of Lithuanian adult population were in line with the recommended daily intakes, while the intakes of phosphorus and copper were higher than the recommended daily intakes. Inadequate intakes in the diet of Lithuanian adults were observed for potassium, calcium, chromium, iron, selenium, and iodine. The median intake of sodium chloride (salt) for Lithuanian adults was determined to be 5.9 g/d which is in line with the recommended daily intake of 5–6 g/d.

Table 6 shows the results of the total mineral intake and the intake according to respondents’ gender and age. As for all minerals, with the exception of calcium, men tended to have statistically higher intakes than women. While analysing the differences in mineral intake with respect to respondents’ age, it was found that the respondents from the youngest age group, compared to older respondents, had a statistically lower intake of chromium and iodine. Women, compared to men, as well as younger respondents, compared to older, had a statistically lower intake of sodium chloride.

**Table 6.** Daily mineral intakes by gender and age (median (25^th^ and 75^th^ percentiles))

Minerals	Total	Gender			Age group (years)			
		Men	Women	*P-value*	19–34	35–49	50–64	*P-value*
Magnesium (mg/day)	276.6 (202.1–381.2)	308.7 (224.8–409.1)	254.0 (188.2–346.8)	<0.0001	272.7 (199.4–374.9)	280.0 (199.2–384.5)	279.8 (206.1–385.4)	0.276
Phosphorus (mg/day)	1044.0 (754.6–1370.1)	1161.7 (849.1–1509.7)	926.8 (698.7–1236.3)	<0.0001	1004.5 (728.4–1337.6)	1057.3 (768.4–1400.3)	1054.8 (775.9–1373.0)	0.084
Potassium (mg/day)	2458.5 (1826.4–3292.8)	2705.2 (2000.5–3645.7)	2263.3 (1737.6–2988.8)	<0.0001	2406.7 (1789.1–3221.3)	2486.5 (1829.8–3350.9)	2473.7 (1884.3–3299.1)	0.087
Calcium (mg/day)	522.8 (347.0–757.2)	561.6 (377.7–850.3)	492.4 (324.3–684.4)	<0.0001	505.0 (332.4–760.2)	531.4 (363.6–747.2)	542.4 (349.7–767.1)	0.208
Chromium (μg/day)	29.4 (16.9–45.5)	33.1 (19.5–50.3)	26.7 (15.5–41.1)	<0.0001	27.1 (15.3–41.9)	31.1 (17.3–48.4)	30.8 (19.5–46.7)	<0.0001
Manganese (mg/day)	2.9 (1.7–4.6)	3.1 (1.9–5.1)	2.7 (1.6–4.2)	<0.0001	2.8 (1.7–4.6)	2.9 (1.8–4.7)	2.9 (1.8–4.5)	0.254
Iron (mg/day)	9.8 (7.0–13.6)	11.2 (8.0–15.6)	8.8 (6.4–11.9)	<0.0001	9.6 (6.6–13.0)	10.0 (7.1–13.8)	9.7 (7.2–13.6)	0.177
Copper (mg/day)	1.6 (1.0–2.2)	1.7 (1.2–2.4)	1.4 (1.0–2.0)	<0.0001	1.5 (1.0–2.1)	1.6 (1.0–2.3)	1.6 (1.1–2.3)	0.162
Zinc (mg/day)	8.4 (5.8–11.5)	9.6 (6.6–13.3)	7.5 (5.2–10.2)	<0.0001	8.1 (5.6–11.4)	8.5 (5.8–11.5)	8.5 (6.0–11.4)	0.419
Selenium (μg/day)	27.8 (26.0–30.9)	34.6 (32.4–37.9)	21.9 (20.3–24.7)	<0.0001	22.0 (20.4–24.8)	32.6 (30.8–35.8)	29.4 (27.6–32.6)	0.332
Iodine (μg/day)	18.8 (2.3–39.9)	21.1 (2.6–45.1)	16.4 (2.1–36.9)	<0.0001	17.0 (2.1–36.1)	18.4 (2.4–43.6)	20.0 (3.8–41.7)	0.019
Sodium chloride (g/day)	5.9 (4.1–8.6)	7.0 (4.8–9.8)	5.3 (3.7–7.3)	<0.0001	5.6 (3.8–8.2)	6.4 (4.3–8.8)	6.1 (4.3–8.8)	<0.0001

Table 7 shows the results of mineral intake according to the respondents’ living area and degree of education. Respondents living in rural areas, compared to those, living in urban areas, had a statistically higher intake of sodium chloride (salt). The intakes of magnesium, phosphorus, and potassium were statistically lower in respondents with a degree of primary education, compared to those, who have attained a higher degree of education. Respondents with a degree in college or vocational education had a statistically higher intake of chromium compared to those with a degree in secondary education. The intake of sodium chloride (salt) was determined to be statistically higher in respondents with college, vocational education in comparison to respondents with secondary or tertiary education.

**Table 7.** Daily mineral intakes by the living area and degree of education (median (25^th^ and 75^th^ percentiles))

Minerals	Living area			Degree of education				
	Urban	Rural	*P-value*	Primary	Secondary	College, vocational	Tertiary	*P-value*
Magnesium (mg/day)	278.2 (202.5–381.1)	271.8 (199.1–381.6)	0.661	220.6 (172.0–364.4)	270.7 (188.4–370.1)	283.8 (208.1–391.6)	276.7 (205.9–381.2)	0.003
Phosphorus (mg/day)	1044.0 (760.5–1361.0)	1044.6 (724.3–1405.8)	0.694	900.3 (588.7–1220.3)	1024.0 (722.7–1405.8)	1084.2 (784.7–1412.7)	1012.3 (755.2–1335.6)	0.003
Potassium (mg/day)	2466.6 (1827.3–3318.1)	2423.8 (1825.6–3318.1)	0.821	1921.8 (1521.3–2811.4)	2392.0 (1758.2–3304.5)	2563.9 (1921.9–3411.3)	2439.4 (1820.0–3233.3)	0.002
Calcium (mg/day)	524.7 (347.1–757.2)	513.5 (346.9–757.7)	0.993	536.5 (352.1–725.1)	515.2 (346.1–779.3)	544.8 (357.4–807.4)	515.3 (344.0–730.9)	0.084
Chromium (μg/day)	29.3 (16.6–44.9)	29.5 (18.5–47.5)	0.075	23.4 (15.9–37.4)	27.6 (15.6–42.3)	33.6 (19.4–48.8)	29.2 (16.6–44.0)	<0.0001
Manganese (mg/day)	2.9 (1.8–4.6)	2.7 (1.6–4.7)	0.239	1.6 (1.4–4.0)	2.8 (1.7–4.8)	3.0 (1.8–4.6)	2.8 (1.7–4.6)	0.133
Iron (mg/day)	9.8 (7.1–13.6)	9.6 (6.6–13.4)	0.281	9.2 (5.7–11.1)	9.6 (6.6–13.8)	10.1 (7.2–13.9)	9.7 (7.0–13.3)	0.094
Copper (mg/day)	1.5 (1.1–2.2)	1.6 (1.0–2.3)	0.551	1.5 (0.9–2.3)	1.5 (1.0–2.2)	1.7 (1.1–2.3)	1.5 (1.0–2.2)	0.421
Zinc (mg/day)	8.4 (5.8–11.4)	8.4 (5.5–11.6)	0.893	8.4 (6.2–10.5)	8.4 (5.6–11.5)	8.5 (6.0–11.6)	8.2 (5.6–11.3)	0.218
Selenium (μg/day)	27.0 (25.3–30.0)	26.9 (24.9–30.7)	0.462	27.5 (25.9–30.5)	28.4 (26.6–31.6)	30.4 (28.6–33.9)	25.0 (23.3–27.8)	0.273
Iodine (μg/day)	18.4 (2.4–39.2)	18.9 (2.1–45.1)	0.449	25.1 (1.2–62.3)	16.9 (2.1–38.0)	20.0 (3.3–39.3)	18.0 (2.2–40.3)	0.346
Sodium chloride (g/day)	5.8 (4.0–8.3)	6.6 (4.5–9.7)	<0.0001	6.4 (5.1–8.5)	5.9 (4.0–9.1)	6.5 (4.6–9.3)	5.6 (3.9–8.2)	<0.0001

## Discussion

Evidence shows that a healthy diet has a positive influence on health and promotes the prevention of common non-communicable diseases (NCDs). Several NCDs, such as cardiovascular diseases, type 2 diabetes, some cancers, and others, are associated with unhealthy diets.^(8,9)^ Despite the fact that a healthy diet is recognized as a very important determinant of disease risk, studies show that the dietary intakes of residents of Lithuania and other countries still does not fully comply with the recommended daily intakes..^(10)^

The energy intake of the adult Lithuanian population was determined to be 1691 kcal and is similar to the energy intake of adults from other Baltic states, however, the energy intake of Lithuanian adults is lower than the energy intake of adults from Northern and Western Europe regions.^(3,11,12)^ Our study revealed that men had a statistically higher intake of energy and this is comparable to what has previously been reported in other population-based surveys.^(11,13)^

Proteins are necessary for development and growth as well as the production of essential amino acids. Furthermore, with an aging world population, consuming enough protein in the diet is a crucial dietary component for preventing disorders like sarcopenia.^(8,9)^ Our study showed that in the diet of Lithuanian adults proteins provide the recommended amount of energy (14.7 E%) and these results are in line with the data obtained by studies carried out in the Nordic and Baltic countries where the intake of protein differs from 14 to 19 E%.^(14)^

Studies show that excessive fat intake is a risk factor for unhealthy weight gain and many NCDs. However, our study showed that the fat intake of the adult Lithuanian population was 42.7 E% and it is one of the highest in Europe since fat intake in Nordic countries typically does not exceed 40 E%.^(14)^ It is important to note that previously carried out studies of nutrient intakes of Lithuanian adults showed similar results for percentage of energy intake from fats, for instance, in the 2014 national study fats contributed 41.7% of energy, and in the 2007 study – 43.2%. One of the possible explanations for high energy intake from fats in the diet of Lithuanian adults could be the reason that Lithuanians tend to frequently consume foods that are high in fat, such as meat, milk, and their products and low fat versions of these products are chosen more rarely.^(15,16)^ It is recommended to limit intake of saturated fats to less than 10% of total energy intake because limiting the intake of saturated fats has been proven to be beneficial for health since it reduces the risk of many cardiovascular diseases.^(17)^ However, our study showed that the intake of saturated fats for Lithuanian adults was 13.7 E% and our findings are similar to those published by studies carried out in other Baltic and Nordic countries where the intake of saturated fats ranges from 13.0 to 15.0 E%.^(14)^

In the previously carried out national studies of nutrient intakes of Lithuanian adults the energy intake from carbohydrates has always been established to be below the lower range of recommended intake (45 E%). It was determined to be 44.4 E% in the 2014 study and 41.1 E% in 2007 study.^(15,16)^ Our study showed that the energy intake from carbohydrates in the diet of Lithuanian adult population was 41.1% and, as in previous studies, it remained below the lower range of the recommended intake. Also, our study showed that women had a higher energy intake from carbohydrates compared to men, which is in line with the findings of other studies carried out in European countries.^(14,18)^

Many adverse health effects including unhealthy weight gain, dental caries or diabetes are associated with excessive intake of sugars.^(8)^ Our study has demonstrated that the median energy intake from sugars in the diet of Lithuanian adults was 9.8 E% and it is one of the lowest among other European countries. However, there are differences among countries when comparing the amounts of energy intake from sugars. For example, in Spain, the energy intake from sugars for adults is 16.7 E%, while in the Netherlands it is 19.5 E%.^(12,19)^ Gender had a significant effect on the energy intake from sugars: women had a statistically higher energy intake from sugars which exceeded the recommended daily intake. Higher energy intake from sugars for women, compared to men, was also observed in other population-based studies.^(20)^

Scientific evidence shows that dietary fibre is crucial for a healthy and balanced diet. Dietary fibre is proven to be important for preventing a number of NCDs, including diabetes, cancer, cardiovascular diseases, and others.^(21)^ Since the first national food consumption and dietary intake study of Lithuanian adult population carried out in 1997, the intake of dietary fibre in the diet of Lithuanian adults has been shown to be insufficient. In 1997 study it was 16.2 g/d, in 2014 study – 15.7 g/d, and our study showed that the intake of dietary fibre remained insufficient – 15.1 g/d.^(15,16)^ The low intake of dietary fibre and the fact that men had a higher intake of dietary fibre compared to women, which was observed in our study, is in accordance with studies carried out in other European countries.^(3)^

The study of the adult Lithuanian population diet showed that the intakes of vitamin A, vitamin E, thiamine, riboflavin, vitamin B6, and vitamin B12 were in adherence or almost in adherence to the recommended intakes. The intakes of these vitamins range across Europe, for example, the intake of vitamin A differs from 692 μg RE/d in the Czech Republic to 1200 μg RE/d in France.^(10)^ The intake of vitamin B6 in Lithuanian adults is similar to the intake of vitamin B6 in other European populations which is between 1.5 mg/d to 2.0 mg/d.^(14,22)^

Insufficient intakes in our study were found for vitamin K, vitamin D, niacin, vitamin C, pantothenic acid, and biotin. Vitamin deficiencies can cause many adverse health effects so a healthy diet with adequate intakes of vitamins ensures the prevention of these adverse health effects. Our study determined that the intake of vitamin D in Lithuanian adults was only 5.2 μg/d which is only half of the recommended intake of 10 μg/d. Several health benefits are associated with an adequate intake of vitamin D, including the health of bones and the proper functions of muscles, nerves, and the immune system.^(23)^ Vitamin D intakes vary across Europe from 4.8 μg/d in Denmark to 11.5 μg/d in Finland.^(14)^

Inadequate intakes in the diet of Lithuanian adults were observed for potassium, calcium, chromium, iron, selenium, and iodine. When comparing the intakes of these minerals among the population of Lithuanian adults and other European adult populations, it was observed that Lithuanian adults had lower intakes of these minerals than adults from other European populations.^(10,23,24)^ Selenium is an essential mineral for the normal function of the cardiovascular, endocrine, and immune systems, therefore, adverse health conditions, including type 2 diabetes, thyroid autoimmune disease and others, are associated with selenium deficiency.^(25)^ It is recommended that Lithuanian adults should consume 50–60 μg of selenium per day (depending on gender and age), however, our study showed that the median intake of selenium is only half of the recommended norm, as it is 27.8 μg/d. Selenium intake in Lithuanian adults is one of the lowest in Europe since in other European countries it differs from 46 μg/d in Sweden to 78 μg/d in Finland.^(14)^ Iodine intakes in the adult Lithuanian population were determined to be 18.8 μg/d which is below the recommended amount. In order to protect public health against the ailments caused by the Lithuanian geographical location, such as iodine deficiency, table salt should contain 20–40 mg/kg of iodine when used in food production to compensate for iodine deficiency.^(26)^

Our study showed that sodium chloride (salt) consumption of Lithuanian adults was 5.9 g/d and it complied with the recommended daily intake. Salt intake differs across Europe from 5.4 g/d in Estonia to 8.2 g/d in Austria.^(3,14)^ Significant differences were observed between salt intakes in women and men – women had a significantly lower sodium chloride intake than men which is in line with other population-based surveys.^(3,22)^

The main advantage of this study is that it uses data from a nationally representative survey and data was collected by well-trained interviewers. However, this study has several limitations. First, a single 24-h recall was used in this study and it is known to have a high within-person variability. However, it is frequently used to estimate food and nutrient intakes in a large group of participants, since multi-day recalls place more of a burden on respondents and can eventually increase drop-out rates. Second, since this study was a cross-sectional look at the diet of Lithuanian adults, results should be interpreted with caution, also there is no accounting for seasonality in food consumption. Lastly, the current paper does not describe foods consumed from which nutrient intakes were calculated, however, the authors are planning to report this in the future publications.

## Conclusions

In conclusion, study showed that intakes of majority of nutrients in the diet of the adult Lithuania population are not in compliance with the recommended daily intakes. Continuous nationally representative studies into food consumption and nutrient intake of the adult Lithuanian population must be carried out to assess the changes in the population’s diet and the effectiveness of policies aimed at promoting healthy diets.

## Abbreviations

**E%:** percentage of the total energy intake; **EU:** European Union; **NCD:** non-communicable diseases; **RDI:** recommended daily intake.
